# Effect of Adding *Matricaria recutita* L., *Cymbopogon citratus,* or *Mentha piperita* L. Extracts to Fermented Orange Beverage: Sensory Evaluation, Physicochemical Characterization, and Prediction of Toxic Risks and Biological Activity In Silico

**DOI:** 10.3390/foods12020243

**Published:** 2023-01-05

**Authors:** Laura Gizele Mascarin, Fernanda Wouters Franco, Rafaela Castro Dornelles, Kássia Caroline Figueredo, Roberta Oliveira Santos, Liliane de Freitas Bauermann, Tatiana Emanuelli, Sabrina Somacal, Cláudia Kaehler Sautter

**Affiliations:** 1 Department of Food Technology and Science, Rural Sciences Center, Federal University of Santa Maria, Santa Maria 97105-900, RS, Brazil; 2 Department of Physiology and Pharmacology, Health Sciences Center, Federal University of Santa Maria, Santa Maria 97105-900, RS, Brazil; 3 Graduate Program in Industrial Systems and Processes, University of Santa Cruz, Santa Cruz do Sul 96815-900, RS, Brazil

**Keywords:** antioxidants, *Cymbopogon citratus*, fermented beverage, functional beverage, gastroprotective, *Matricaria recutita* L., *Mentha piperita* L., phenolic compounds

## Abstract

Fermentation is an important tool in producing functional beverages through agro-industrial wastes, and medicinal and aromatic plants due to the specific content of bioactive molecules. Therefore, this study evaluated the contribution of *Matricaria recutita* (chamomile), *Cymbopogon citratus* (lemongrass), or *Mentha piperita* (peppermint) extracts to the phytochemical profile and potential biological effects of a functional fermented orange beverage in vitro and in silico. The concentrations of aromatic herbal extracts that yielded the best sensory performance for fermented beverages were selected for analyses that involved characterizing the fermented beverages. The beverages that received the extracts (2%) had the highest phenolic and flavonoid content and antioxidant potential compared to the control. Hesperidin (124–130 mg L^−1^), narirutin (66–70 mg L^−1^), chlorogenic (11–16 mg L^−1^), caffeic (5.3–5.5 mg L^−1^), and ferulic (1–1.7 mg L^−1^) acids were found in the different formulations. The in silico analysis suggested that the evaluated compounds do not present a toxicity risk (mutagenicity, carcinogenicity, hepatotoxicity, and ability to penetrate the blood–brain barrier). Additionally, they can contribute to the biological effects of therapeutic importance, such as antioxidant, gastroprotective, and anti-ulcerative properties, and the *Mentha piperita* L. extract presented the greatest potential among the evaluated herbs for use in functional fermented beverages.

## 1. Introduction

Consumer dietary habits have drastically changed in recent decades and functional beverages now have a strong position in the market. Most of these beverages are produced using simple processes that use fruits, cereals, legumes, nuts, and food product waste, among other things [1,2]. Recently, wine, one of the most consumed and preferred alcoholic beverages, has been identified as a functional beverage [3]. In addition to the grape, wine can be produced from any sugary fruit as long as it is designated as fermented fruit [4]. In the last decade, various studies have reported the production of fruit wine and its possible therapeutic properties [4,5]. The main source of these beverages’ beneficial potential is phenolic compounds [2,3]. After consuming foods rich in phenolic compounds, such as functional beverages, the colon is the main site of microbial fermentation. Phenolic compounds are transformed into phenolic acids or lactone structures by intestinal microbiota, which produces metabolites with biological and antioxidant activity, and evidence suggests those metabolites have health benefits for humans [2,6].

Oranges are an abundant source of vitamin C and have considerable amounts of sugar, minerals, and bioactive compounds such as carotenoids, phenolic compounds, and terpenoids [5,7]. Thus, their use as raw materials to produce functional beverages is an attractive option to add value to the product and diversify the market, and a solution to minimizing the losses of these fruits in the fields or during transportation [1]. In addition, Brazil is considered the largest producer of oranges [8], and its different fruiting periods enable various harvests, thus avoiding the concentration of crops and reducing production costs throughout the year [8].

The use of medicinal and aromatic plants in the production of functional beverages has become increasingly popular due to the specific content of structurally diverse bioactive molecules with numerous confirmed health benefits and specific sensory properties [9]. The aromatic features of *Matricaria recutita* L. (chamomile), *Cymbopogon citratus* (lemongrass), and *Mentha piperita* L. (peppermint) are mainly related to volatile compounds of essential oils. However, the presence of non-volatile compounds, including phenolics, also contribute to specific sensory and beneficial properties [10]. Phytochemical profiling of medicinal and aromatic plants containing specific and complex mixtures of bioactive molecules provides numerous opportunities to develop new categories of functional beverages.

The herbs *Matricaria recutita* L., *Cymbopogon citratus*, and *Mentha piperita* L. have anti-inflammatory, antioxidant, antiseptic, gastroprotective, and many other properties attributed mainly to their polyphenols and essential oil components [11,12,13]. The beneficial effects of *Matricaria recutita* are related to the presence of phenolic compounds (e.g., apigenin or hydroxycinnamic acid derivatives) and the essential oil components (e.g., chamazulene, farnesene, *α*-bisabolol, and its oxides) [14]. Among the *Cymbopogon citratus* volatile compounds, the citral (mixture of terpenoids and geranial), myrcene, geraniol, citronellol, and α-oxobisabolene stand out [13]. In the case of *Mentha piperita*, the emphasis is on *d*-carvone, limonene, menthone, menthol, and pulegone, and among non-volatile compounds phenolic acids such as chlorogenic, caffeic and rosmarinic acids, and flavonoids, including luteolin, naringenin, and hesperetin derivatives [11]. This diverse profile of compounds allows them to be potentially included in beverages [9], generating a differentiated product.

In this context, identifying the main biologically active compounds with antioxidant properties is of great interest to the functional food and beverage industry. In addition, new global trends of concern with biodiversity and ideas of sustainable development have brought the importance of using in silico methods to precede experimental studies in vivo, reducing production time and costs [15]. In silico analysis, through the evaluation of the structural analysis of compounds, can be used to estimate their toxicity and/or predict their biological activity profile, among other effects [16].

Thus, this study aims to analyze the contribution of *Matricaria recutita* L., *Cymbopogon citratus*, and *Mentha piperita* L. extracts in the phytochemical profile and potential biological effects of a functional fermented orange beverage.

## 2. Materials and Methods

### 2.1. Chemicals

Milli-Q water (Millipore, Bedford, MA) was used in all experiments. 2,2-Diphenyl-1-picrylhydrazyl hydrate (DPPH), catechin (98%), Folin–Ciocalteu reagent (2N), chlorogenic acid (95%), caffeic acid (98%), ferulic acid (99%), gallic acid (98%), protocatechuic acid (95%), synergic acid (95%), synaptic acid (95%), t -cinnamic acid (95%), and rutin (94%) were obtained from Sigma-Aldrich (St. Louis, MO, USA). Hesperidin (98%), naringenin (98%), narirutin (98%), apigenin (98%), and luteolin (98%) were purchased from Cayman Chemical Company (Ann Arbor, MI, USA). HPLC-grade methanol and acetic acid were acquired from Sigma-Aldrich (St. Louis, MO, USA) and used in the mobile phase. All other chemicals were of analytical grade.

### 2.2. Experiment 1—Production of Fermented Orange Beverage and Choice of Aromatic Herb Concentrations

#### 2.2.1. Raw Material: Oranges and Aromatic Herbs

In order to produce the fermented orange beverages, 200 kg of “Valencia” oranges (2018 harvest) were purchased in the city of Mata, southern Brazil. The aromatic herbs—*Matricaria recutita* L. (flowers), *Cymbopogon citratus* (leaves), and *Mentha piperita* L. (leaves)—were purchased locally and in dry form, which is suitable for making teas.

#### 2.2.2. Fermented Orange Beverage Production with the Addition of Aromatic Herbal Extracts

The oranges were selected and sanitized (200 ppm sodium hypochlorite). The juice was extracted by performing a latitudinal cut on the oranges, which were squeezed in an industrial extractor (JL Colombo^®^ 650W, Itajobi, Brazil). The juice was filtered to remove the solid residue, and a yield of 39% was obtained.

The total soluble solids (TSS) of the juice were determined by refractometry and the pH was measured with a digital pH meter (Digimed^®^ model DM–22, São Paulo, Brazil) [17].

The orange juice (must) was prepared for fermentation by adding 6% sodium metabisulfite (70 ppm) and left to rest for 60 min. Then, 230 g L^−1^ of sugar (26 °Brix) was added, homogenized, and the pectinolytic enzymes (Lafazin Extract^®^, 3 g hL^−1^; NutriStart^®^, 40 g hL^−1^) were added.

The yeast *Saccharomyces cerevisiae* (Blastosel Delta^®^, 40 g hL^−1^) was inoculated and the alcoholic fermentation was carried out in 20 L polyethylene kegs at a controlled temperature (16 ± 2 °C), and with decreased and stabilized TSS to determine the endpoint of fermentation.

After fermentation, the temperature was maintained at 5 °C for 48 h to separate the yeasts and other solids. The must was racked and filtered, and 50 ppm of 6% sodium metabisulfite was added and kept stabilized for three months with a controlled temperature of 16 ± 2 °C.

The hydro-alcoholic extracts of aromatic herbs (50% EtOH/H_2_O) were prepared from equal volumes of 96 °GL cereal alcohol and distilled water. Then, 100 g of dry matter from each plant was weighed and placed in maceration in 1 L of 50% hydroalcoholic solution, which was the volume necessary to fully cover the dry matter, resulting in an extract concentration of 10 g% [9]. The herbs were kept in infusion in dark bottles at room temperature for 14 days to optimize the extraction of compounds [18]. Afterward, the extracts of *Matricaria recutita* L., *Cymbopogon citratus*, or *Mentha piperita* L. were added to the fermented beverage, thus forming 16 treatments: 1 control and 5 concentrations (0.5, 1.0, 2.0, 3.0, and 4.0%) (*v/v*) for every aromatic herb. The mixture remained stabilized for 2 months, in the dark, and at a controlled temperature of 16 ± 2 °C [9].

#### 2.2.3. Sensory Evaluation of Functional Fermented Orange Beverages

Sensory evaluation was carried out at the Federal University of Santa (Rio Grande do Sul State, southern Brazil) at the Integrated Center for Development and Laboratory Analysis (NIDAL), in a laboratory with partitioned booths under white light. Participants were fully informed of the experimental protocol and the possible risks and discomforts of the investigation before giving their written informed consent. The study was conducted according to the Declaration of Helsinki, and the study protocol was approved by the institutional ethics committee (CAAE number:10027519.3.0000.5346, no. 3.257.616).

Twenty-five untrained panelists (men and women ages 18–60 years) were selected to participate based on their preference for wines, interest, and availability. The samples (20 mL) were randomly chosen and served chilled (7 ± 1 °C) in colorless vessels numbered with three random digits. Mineral water and water-and-salt biscuits were provided to clean the palate and powdered coffee for olfactory fatigue.

The evaluations were performed on three different days (day 1 = *Matricaria recutita* L., day 2 = *Cymbopogon citratus*, and day 3 = *Mentha piperita* L.). The samples were evaluated by an affective acceptance test using a 7-point hedonic scale (1 = disliked extremely, 2 = disliked a lot, 3 = disliked a little, 4 = disliked, 5 = liked, 6 = liked a lot, 7 = liked extremely), in which the color, aroma, and flavor attributes were judged. In the order preference test, the samples were ordered from the least preferred to the most preferred. The result is the sum of the orders, where higher values for the samples represent greater preference. The purchase intention test was carried out using a 5-point scale (1 = would certainly buy, 2 = would likely buy, 3 = may or may not buy, 4 = would likely not buy, 5 = would certainly not buy) [19].

For each aromatic herbal extract tested, the concentration that yielded the best sensory performance for fermented beverages was selected for subsequent analyses.

### 2.3. Experiment 2—Physicochemical Characterization and Bioactive Parameters of Functional Fermented Orange Beverages

The best concentration of the set of herbs in Experiment 1 led to the following treatments for Experiment 2: fermented control; fermented beverage with *Matricaria recutita* L. (MR); fermented beverage with *Cymbopogon citratus* (CC); and fermented beverage with *Mentha piperita* L. (MP).

#### 2.3.1. Physicochemical Characterization

All physicochemical characterization analyses were carried out in triplicate, with methodology indicated to meet the parameters of Brazilian legislation [17]. The alcohol content (% ethanol) was determined in a Gilbertini^®^ enochemical distiller (Novate Milanese, Italy). Total acidity (mEq L^−1^ of citric acid) was determined by titrimetry. Volatile acidity (mEq L^−1^ of acetic acid) was determined by steam dragging in a Gilbertini^®^ enochemical distiller. The fixed acidity (mEq L^−1^ of citric acid) was determined by converting volatile acidity into fixed acidity, considering the gram-equivalent values (Eq g^−1^) of citric acid per Eq g^−1^ of acetic acid. The pH of the samples was measured through digital reading, carried out in a pHgameter (Digimed^®^ DM—22, São Paulo, Brazil). Reducing sugar determination (g L^−1^) was conducted according to Lane and Eynon’s redox titration method. The reduced dry extract content (g L-1) was determined as the dry residue’s weight obtained after the volatile compounds’ evaporation.

#### 2.3.2. Total Phenolic and Total Flavonoid Content, and Antioxidant Potential

Total phenolic content (TPC) was determined by the colorimetric method with the Folin–Ciocalteau reagent [20] using a spectrophotometer at a wavelength of 765 nm. Quantification was performed using a calibration curve (0 to 80 mg L^−1^; y = 105.19x + 1.4331; R² = 0.989), and the results were expressed in milligrams of gallic acid equivalent per liter of the sample (mg GAE L^−1^).

Total flavonoid content (TFC) was determined by spectrophotometry with absorbance at a wavelength of 510 nm [21]. Quantification was performed using a calibration curve (0 to 200 mg L^−1^; y = 305.79x−3.0507; R² = 0.999), and the results were expressed in milligrams of catechin equivalents per liter of sample (mg CAE L^−1^).

Antioxidant capacity was measured by eliminating DPPH radicals [22] in the samples of fermented orange beverages composed of aromatic herb extracts and samples of crude herbal extracts with a reading at 517 nm after 4 h of incubation in the dark. The effective sample concentration required to eliminate DPPH radicals by 50% (IC_50_ value) was obtained by linear regression analysis and represented the sample concentration in relation to the corresponding elimination effects. The lower the IC_50_ values, the greater the antioxidant activity.

The antioxidant potential of all beverages and crude extracts was also determined as their potential to protect against fluorescein oxidation by the peroxyl radical generated through the thermal degradation of AAPH using the oxygen radical absorbance capacity (ORAC) method [23]. Briefly, the reaction was carried out at 37 °C in 75 mM phosphate buffer (pH 7.4), where 25 μL of sample or Trolox and 150 μL of fluorescein (81 nM) were placed in the well of the black 96-well microplates. The mixture was pre-incubated for 10 min at 37 °C. The AAPH solution (25 μL; 152 mM) was rapidly added using a multichannel pipet, and the fluorescence was recorded every minute for 90 min. Fluorescence (λexc = 485 nm and λem = 528 nm) was measured using a Synergy H1 plate reader (Agilent, USA). The ORAC values were calculated by a regression equation obtained with the area under curve (AUC) of the fluorescein decay. This analysis was expressed as µmol of Trolox equivalents per liter of sample (µmol TE L^−1^).

#### 2.3.3. HPLC Analysis of Phenolic Compounds

The samples’ main phytochemicals with antioxidant potential were by high-performance liquid chromatography analysis combined with diode array detection analysis (HPLC-DAD), according to the adapted method [24]. The compounds were separated using liquid chromatography (SHIMADZU, Kyoto, Japan) composed of a pump (model LC-20AT), an automatic injector (SIL-20A), a diode array detector (DAD SPD-M20A), and a communicator (CBM 20A). The separation was controlled by the software LC SP1.

The Agilent Eclipse Plus C18 column (4.6 × 150 mm, 5 µm) was used with gradient elution (flow rate of 0.8 mL min^−1^) using two mobile phases (A and B). Mobile phase A consisted of 2% acetic acid in the water, while mobile phase B consisted of only UV/HPLC methanol. The injection volume of the samples was 40 µL, and the detection was monitored in a photodiode system at wavelengths between 230–400 nm for 55 min. Before injection, the samples and mobile phases were filtered through a 0.45 µm hydrophilic nylon membrane.

Compound identification was performed by comparing the retention time in the samples and visible UV absorption spectrum with solutions of authentic standards. The standards used were: gallic acid, protocatechuic acid, synergic acid, synaptic acid, t-cinnamic acid, chlorogenic acid, caffeic acid, ferulic acid, rutin, narirutin, hesperidin, naringenin, apigenin, and luteolin. The identified compounds were quantified by calibration curves obtained by preparing solutions at 1000 mg L^−1^ in MeOH UV/HPLC with the standards. The points used were: 5.0, 10.0, 25.0, 35.0, and 50.0 mg L^−1^.

The contents of the quantified substances were calculated from the line equation and the results were expressed in milligrams equivalent to each standard per milliliter of each sample (mg L^−1^).

#### 2.3.4. Prediction of Toxic Risks In Silico

A computer simulation study was carried out to estimate possible toxicity risks in the main compounds identified by HPLC-DAD using four online computer programs: admetSAR server (https://www.simulations-plus.com/software/admetpredictor (accessed on 15 December 2020)), pkCSM platform (http://biosig.unimelb.edu.au/pkcsm (accessed on 16 December 2020)), Protox (http://tox.charite.de/protox_II (accessed on 15 December 2020)), and Lazar (http://lazar.in-silico.ch/predict (accessed on 15 December 2020)). The toxicological safety comprised the predicted risk for the following toxic effects: mutagenicity, carcinogenicity, hepatotoxicity, blood–brain barrier permeability, and acute oral toxicity. Side effects were interpreted and expressed as: (+) at risk, (-) not at risk, and (nr) not reported.

Acute oral toxicity was classified based on the four categories of the United States Environmental Protection Agency, which divides the compounds according to their LD_50_ values (mean lethal dose). Category I contains compounds with LD_50_ values ≤ 50 mg kg^−1^, Category II contains compounds with LD_50_ values > 50 mg kg^−1^ and < 500 mg kg^−1^, Category III includes compounds with LD_50_ values ≥ 500 mg kg^−1^ and < 5000 mg kg^−1^, and Category IV consists of compounds with LD_50_ values ≥ 5000 mg kg^−1^. (MP).

#### 2.3.5. Prediction of Biological Activity In Silico

The possible biological effects of the main compounds identified by HPLC-DAD were predicted with computer simulations using the platform Way2Drug PASS online (http://www.way2drug.com (accessed on 17 December 2020)).

The predicted results were expressed as a percentage of probably active (Pa) and probably inactive (Pi). Results with Pa greater than 70% indicate a high probability of pharmacological activity [25].

The criterion for selecting the main biological effects was the gastroprotective potential and/or healing potential of the identified compounds. The selected effects were antihemorrhagic, antioxidant, anti-free-radical, anti-inflammatory, and anti-ulcerative properties, gastritis treatment, mucous membrane protection, and vasoprotection.

### 2.4. Statistical Analysis

The analyses were performed in triplicate, and the results were submitted to Pearson’s correlation analysis and analysis of variance (ANOVA), and the means compared by Tukey’s test at 5% probability. All statistical analyses were performed using the Statistica 10.0 software. In the sensorial analysis, an experimental design of completely randomized blocks was used and analyzed by the Friedman method.

## 3. Results

### 3.1. Sensory Evaluation

Different sensory tests were conducted to determine the most acceptable concentration of aromatic plant extract added to the fermented orange beverage.

The attributes “color” and “aroma” presented good evaluations (Table 1), which indicates that all extracts, in all concentrations, pleased the panelists. Nevertheless, they did not present significant differences (*p* > 0.05) between the concentrations of the samples; therefore, they cannot be considered criteria for selecting the best concentration.

Regarding “flavor,” all extracts showed a significant difference (*p* < 0.05) between the lowest concentration (0.5%) and the highest concentration (4.0%). These data revealed that the panelists preferred the fermented orange beverage with lower concentrations of aromatic herbal extracts (between 0.5 and 2.0%). Moreover, the panelists considered both beverages with CC and MP to be “very pleasant” and “very refreshing” (data not shown).

In the order preference test (Table 1), there was a significant difference (*p* < 0.05) in all extracts regarding the highest concentration (4.0%), which was indicated by the majority of the panelists as the least preferred. These data corroborate the assessments presented in the acceptance test.

For the consumption and purchase intention test (Table 1), the concentration of 4.0% showed a significant difference (*p* < 0.05) in all extracts, being indicated as “may or may not buy” and “would likely not buy.” The concentrations of 0.5, 1.0, and 2.0%, despite not showing significant differences (*p* > 0.05), received indications of “would certainly buy” and “would likely buy,” thus corroborating the other observations in the previous tests.

In our studies, the concentration of 2.0% was the highest in all extracts when there was no significant difference (*p* > 0.05) in any of the attributes (color, aroma, and flavor) (Table 1), with the highest concentration being accepted in the order preference test and purchase intention test, and, therefore, the concentration chosen for subsequent evaluations.

### 3.2. Physicochemical Characterization

The physicochemical characterization of the beverages (Table 2) was in accordance with Brazilian legislation [26]. The results showed no significant differences between the control and the other treatments (*p* > 0.05), suggesting that the added aromatic plant extracts did not alter the beverages’ physicochemical parameters.

### 3.3. Total Phenolic and Flavonoid Content, and Antioxidant Capacity

The TPC, TFC, and antioxidant capacity of the extracts and beverages developed in this study can be seen in Table 2. The addition of the MP extracts increased TPC by 8%, indicating a possible increase in the reducing capacity of this beverage. The TPC of the beverages added with MP and CC extracts increased by 5 and 3%, respectively. Therefore, the results of our study show that the extracts contributed to the greater antioxidant potential in the beverages. Notably, despite being widely used to assess total phenolic content, this TPC assay measures the reducing capacity of samples [27].

The addition of aromatic herbal extracts contributed significantly (*p* < 0.05) to TFC. Hence, the MP extracts increased TFC by 101%, MR by 29%, and CC by 12%.

Antioxidant capacity evaluated by DPPH showed significant differences in all samples (*p* < 0.05), both in the crude herbal extracts and fermented orange beverages. The values found in the crude herbal extract had greater antioxidant capacity in the MP extract, as indicated by the lower IC_50_ values, followed by MR and CC. Nevertheless, the ORAC test showed that only the fermented orange beverage with the addition of MP extract had a greater capacity to neutralize the peroxyl radical when compared to the control beverage. These data suggest that the analyzed polyphenols are responsible for the antioxidant capacity of the extracts and corroborate the significant antioxidant capacity in vitro of these compounds, which can capture a wide range of reactive oxygen species. In the orange fermented samples with aromatic herbal extracts added, mainly MP, the antioxidant capacity was even more intense, indicating possible synergistic effects with bioactive compounds from the oranges, fermentation process, and extracts.

According to DPPH, adding the MP extract to the orange beverage helped increase antioxidant capacity by about 17%, followed by CC, which contributed to a 6% increase. The MR extract, however, resulted in a 6% reduction in antioxidant activity compared to the control, showing that the extract interacted with the matrix in the DPPH.

The addition of the extracts contributed to an increase in TPC (r = 0.607; *p* < 0.05) and flavonoids (r = 0.775; *p* < 0.01), where the antioxidant potential in the beverages followed the order MP > MR > CC. This same proportion was observed in the crude herbal extracts, where the evaluation of antioxidant activity using the DPPH method for them showed a positive correlation with the content of phenolics (r = 0.932; *p* < 0.05) and flavonoids (r = 0.673; *p* < 0.01).

### 3.4. Composition of Phenolic Compounds

Of the 14 analytes analyzed in this study, 3 phenolic acids and 5 flavonoids were identified in the samples of crude herbal extracts and fermented orange beverages (Table 3 and Figures S1–S4). The major compounds found in the crude herbal extracts were apigenin in the MR (138.4 mg L^−1^) and MP extracts (132.4 mg L^−1^). Other compounds identified in the MR and MP extracts were ferulic acid (95.2 and 98.7 mg L^−1^, respectively), chlorogenic acid (49.1 and 47.4 mg L^−1^, respectively), and luteolin (20.1 and 17.4 mg L^−1^, respectively). In the CC extract, chlorogenic acid (8.4 mg L^−1^), caffeic acid (3.5 mg L^−1^), and luteolin (3.11 mg L^−1^) were identified.

In the beverage control, the hydroxycinnamic acids found were chlorogenic acid (10.9 mg L^−1^), caffeic acid (5.3 mg L^−1^), and ferulic acid (1.6 mg L^−1^). The flavonoids identified were narirutin (69.7 mg L^−1^), rutin (6.1 mg L^−1^), and hesperidin (130.3 mg L^−1^).

The addition of the herbal extracts in the fermented beverages increased the concentration of chlorogenic acid compared to the control. Moreover, there was a 46% increase in the beverage with MR and 26% in the beverage with CC. The addition of the herbs did not change the concentration of caffeic acid. In the beverage with MP, the ferulic acid decreased by 32% and CC by 39%.

Regarding the major flavonoids, hesperidin was reduced by 4% in the beverage with MP and 5% in the one with CC compared to the control. The additions of MR and MP did not alter the concentration of narirutin. However, it was reduced by 5% with CC compared to the control.

### 3.5. In Silico Toxicity Prediction

The computational model suggested that the evaluated compounds did not present any risks of mutagenicity, carcinogenicity or hepatotoxicity, and could not penetrate the blood–brain barrier (Table 4).

Regarding the acute oral toxicity of the analytes, chlorogenic acid, narirutin, rutin, and hesperidin have median toxicity (LD_50_) for concentrations between 500 and 5000 mg kg^−1^ and are identified as class III. Caffeic and ferulic acids have median toxicity (LD_50_) for concentrations ≥5000 mg kg^−1^ and are identified as class IV. Thus, the beverages with added plant extracts did not have the potential for acute toxicity, since the concentrations of these beverage analytes are lower than the LD_50_ (Table 3).

### 3.6. Prediction of Biological Activity In Silico

To identify possible pharmacological effects, the major phytochemicals in the beverages were analyzed for their different types of predicted biological activity (Table 4).

The pharmacological analysis of chlorogenic acid showed that this compound likely has several biological properties of therapeutic importance (Pa > 70%), including antioxidant activity, free radical scavenging, and mucoprotective agents.

Caffeic acid has the potential to act as a mucoprotective and vasoprotective agent. Ferulic acid is a potential scavenger of free radicals and a mucoprotective and vasoprotective agent. The flavonoids narirutin, rutin, and hesperidin have potential anti-hemorrhagic, antioxidant, and anti-inflammatory activity, free radical scavenging, and vasoprotective effects. Narirutin and hesperidin have potential antiulcerogenic effects.

## 4. Discussion

In recent years, our society began demanding healthier products, drastically changing dietary consumer habits. In this context, functional foods provide health benefits beyond basic nutritional functions, and beverages are by far the most important category [1]. The primary purposes of consuming these beverages are boosting energy, fighting aging, fatigue, and stress, weight management, and targeting specific diseases (e.g., hypercholesterolemia, helping decrease glucose levels, etc.) [2,3]. Nonetheless, producing attractive functional foods due to their sensory characteristics is a permanent challenge in the food industry. Gathering healthy and attractive items in a single food is an even greater obstacle. The extensive list of benefits associated with phenolic compounds, including antioxidant, anticancer, anti-inflammatory, and anti-aging properties, among others, fully justifies their use in the enrichment of various food products. Hence, this study sought to develop a functional fermented beverage composed of aromatic herbal extracts, with olfactory richness perceived by the panelists. The concentration of 2% (*v/v*) was the highest in which the “flavor” attribute stood out in the order preference, and consumption and purchase intention tests. Therefore, all the analyses reported below refer to the beverage developed and added with 2% (*v/v*) of chamomile, lemongrass, and mint extracts.

The physicochemical characterization of produced beverages is a legal requirement for the aspects that will make the product proper or not for commercialization and consumption. According to Brazil’s identity and quality standards, the beverage developed should be designated as “Fermented Fruit Compound” [26]. Our findings showed that the beverage meets all the required legal parameters and has characteristics of being “dry” due to the low content of reducing sugars and “full-bodied,” according to the high results obtained in the analysis of dry extract [26]. To meet legislative requirements and the purpose of the study with gastric ulcers (data not yet published), the developed beverage contains a higher alcohol content (15.9–16.2%) than the fermented ones reported in the literature.

In our study, the fermented beverages with CC and MP were well-accepted at the lowest concentrations. The concentration of 2.0% was the highest and most accepted in the preference ordering test, and the consumption and purchase intention tests. The consumption of fruit-based beverages, fermented or not, rich in phenolic compounds has also been related to healthy diets, such as the Mediterranean diet, and the prevention of chronic diseases since they present antioxidant properties [1,2,5]. Although most phenolic compounds have low bioavailability after digestion, they attain remarkably high concentrations in the gastrointestinal tract [6], where they may exert direct radical scavenging and antioxidant properties. Phenolic content varies according to the cultivar due to the genetic potential of its biosynthesis. One study analyzed citrus juices and reported that the TPC was 784.7 mg L^−1^ for bitter oranges and 106.2 mg L^−1^ for mandarin oranges [7]. In another study with “Valencia” oranges, 571.0 mg L^−1^ of TPC was detected in the fruit [28]. This is the same cultivar utilized in our study in which a TPC of 459.4 mg L^−1^ was observed in the produced beverage. Furthermore, the TPC reported herein is relatively high, as a similar study with “Kozan” oranges reported a 48.7% loss in TPC from orange juice (317.36 mg L^−1^) to orange wine (162.7 mg L^−1^) [29].

The bioactive antioxidant properties of phenolic compounds are especially relevant for gastrointestinal disorders as this site attains the greatest concentrations. Phenolic compounds have demonstrated beneficial effects against gastrointestinal disorders associated with oxidative stress such as gastric ulcers or inflammatory bowel diseases [30]. Polyphenols are essential bioactive molecules with potential gastroprotection, which can prevent lesions of the gastric mucosa and reduce the number and intensity of lesions [12,30]. Among the total polyphenols, flavonoids are the major phenolic compounds in oranges, conferring a wide range of biological activities with potential beneficial effects against cardiovascular diseases, osteoporosis, and cancer [1,5,12].

In beverages with added aromatic herbal extracts, it was possible to verify the synergistic effects of bioactive compounds from the orange and extracts, for TPC and TFC. However, concerning the individually quantified analytes, there were slight differences. The extracts were added to the orange beverage that was already prepared, meaning they did not go through the fermentation process together with the beverage. Originally, the crude extract was in a concentration of 10 g% that, when added to the drinks, passed the concentration of 2% (*v/v*). Thus, it is expected that there will be a dilution of these bioactive compounds in the final product. This was observed with luteolin and apigenin which were present in the crude extracts but were not detected in the fermented ones. Rutin was not found in the extracts, although it was identified in the fermented control. However, adding the herbs was not detected, suggesting the interaction with the extracted matrix.

The chromatographic analysis identified chlorogenic acid, caffeic acid, ferulic acid, narirutin, rutin, and hesperidin. A different study on the composition of orange wine made with the “Kozan” cultivar revealed the presence of chlorogenic acid (4.7 mg L^−1^), caffeic acid (2.6 mg L^−1^), narirutin (21.7 mg L^−1^), and hesperidin (90.6 mg L^−1^) in relatively lower values than those found here with the “Valencia” cultivar, except for ferulic acid (9.9 mg L^−1^) [29]. The alcohol content of this study is high due to it being a fermented fruit compound beverage and is 25% higher than the “Kozan” fermented beverage [29]. This concentration may have favored the extractability of these analytes. The bioactive compound content in the extracts can be influenced by various factors, including the extraction method, climatic, geographic, and cultivar conditions, part of the plant material used, its origin, the processing, and even the particle size [31].

Another point of interest for future studies is predicting the bioactivity of the identified phytochemicals. The in silico methods precede in vivo experimental studies, reducing the time spent and laboratory costs [15]. Oral in vivo administration of the chamomile extract was effective in preventing gastric ulceration in mice and did not produce acute toxicity effects at doses up to 5000 mg kg^−1^ [32]. The beverage prepared here with chamomile extract and other herbs contains a proportion of 2 g% of the product, representing an intake of 100 mg kg^−1^.

Our results of the in silico analysis corroborate other data in the literature that attribute to the tested phytochemical groups a wide number of pharmacological activities such as anti-inflammatory, antioxidant, and gastroprotective activity [11,12,13]. Umre et al. (2015) conducted in silico and in vitro studies [33] and demonstrated the antiulcerogenic potential of ferulic acid, and their in vivo analysis reported reduced gastric secretion by delaying the deterioration of the gastric mucosa in different models of gastric ulcer. Moreover, their findings on in silico coupling allowed them to confirm all the interactions of effects observed in the in vivo assay.

In silico modeling for the aqueous extract of *Achyrocline satureioides* flowers revealed that isoquercitrin, quercetin, and caffeic acid had a low probability of toxic risk [34]. Toxicity tests aim to identify harmful effects caused by substances in humans, animals, plants, or the environment through acute or multiple exposures [35]. They also showed that in silico toxicology, by using computational resources, can organize, analyze, model, simulate, visualize, and predict the toxicity of chemical substances with possible beneficial or adverse effects for therapeutic purposes.

## 5. Conclusions

The addition of *Matricaria recutita*, *Cymbopogon citrates*, or *Mentha piperita* extracts at a 2% level in a fermented orange beverage had the best evaluation in sensory tests and positively influenced the functional characteristics of the fermented orange beverage by increasing the total phenolic and flavonoid content, in addition to improving the antioxidant capacity without altering the physicochemical characteristics of the beverage. Among the evaluated extracts, the *Mentha piperita* proved to have the best characteristics to be added to the functional fermented beverage.

Fermented orange beverages have different bioactive compounds (flavonoids and phenolic acids) in their composition that demonstrate gastroprotective and anti-ulcerative potential through in silico evaluation, indicating potential beneficial properties related to the consumption of these beverages—the properties of which will be investigated in future in vivo complementary studies.

## Figures and Tables

**Table 1:** Sensory evaluation of the functional fermented orange beverage containing increasing aromatic herbal extracts.

Sensory Test	Parameter	Herbal Extract	Concentration of herbal Extract mL.100 mL^−1^				
			0.5	1.0	2.0	3.0	4.0
Acceptance test ^X^	Color	MR	5.72 ^a^	5.63 ^a^	5.64 ^a^	5.63 ^a^	5.64 ^a^
		CC	5.76 ^a^	5.72 ^a^	5.48 ^a^	5.52 ^a^	5.36 ^a^
		MP	5.72 ^a^	5,68 ^a^	5.24 ^a^	5.08 ^a^	5.04 ^a^
	Aroma	MR	5.24 ^a^	5.16 ^ab^	5.32 ^a^	4.72 ^ab^	4.36 ^b^
		CC	5.08 ^a^	5.16 ^a^	5.28 ^a^	5.20 ^a^	4.92 ^a^
		MP	5.04 ^a^	5.08 ^a^	5.36 ^a^	5.32 ^a^	5.16 ^a^
	Flavor	MR	5.28 ^a^	4.44 ^ab^	4.88 ^a^	4.36 ^ab^	3.48 ^b^
		CC	5.12 ^ab^	5.40 ^a^	4.88 ^ab^	4.32 ^ab^	4.08 ^b^
		MP	5.12 ^a^	4.72 ^ab^	4.72 ^ab^	3.84 ^b^	3.92 ^b^
Preference test ^Y^		MR	88 ^a^	88 ^a^	73 ^a^	69 ^a^	57 ^b^
		CC	83 ^a^	82 ^a^	82 ^a^	73 ^a^	59 ^b^
		MP	92 ^a^	90 ^a^	77 ^a^	57 ^b^	59 ^b^
Purchase intention ^Z^		MR	2.56 ^ab^	2.88 ^ab^	2.24 ^b^	3.20 ^ab^	3.48 ^a^
		CC	2.44 ^a^	2.52 ^a^	2.64 ^a^	3.28 ^a^	3.32 ^a^
		MP	2.28 ^b^	2.56 ^b^	3.00 ^ab^	3.60 ^a^	3.60 ^a^

^X^ Different letters in the same line indicate means with significant differences (*p* < 0.05) by the Tukey test (n = 25). ^Y^ Values represent the sum of the orders according to Friedman's method and Newell and MacFarlane's table. Different letters in the same line indicate values with significant differences (*p* < 0.05) by the Tukey test (n = 25). ^Z^ MR *= Matricaria recutita* L.; CC = *Cymbopogon citratus*; MP = *Mentha piperita* L. Acceptance tests were assessed using a 7-point hedonic scale where 1= strongly dislike, 2 = disliked a lot, 3 = disliked a little, 4 = disliked, 5 = liked, 6 = liked a lot, and 7 = strongly like. The preference test assessed sample order from the least preferred to the most preferred, yielding higher values for the most preferred samples. The purchase intention test was assessed using a 5-point hedonic scale where 1 = would certainly buy and 5 = would certainly not buy.

**Table 2:** Physicochemical characterization, total phenolic and flavonoid content, and antioxidant capacity of the functional fermented orange beverage containing aromatic herbal extracts.

Parameters	Crude Extract			Fermented Orange Beverage			
	MR	CC	MP	Control	Fermented + MR	Fermented + CC	Fermented + MP
Physicochemical characterization							
Alcohol (%)	na	na	na	16.2 ^a^	16.1 ^a^	15.9 ^a^	16.2 ^a^
Total acidity (mEq L^−1^)	na	na	na	122.0 ^a^	119.3 ^a^	118.9 ^a^	122.7 ^a^
Volatile acidity (mEq L^−1^)	na	na	na	10.1 ^a^	9.1 ^a^	10.4 ^a^	10.8 ^a^
Fixed acidity (mEq L^−1^)	na	na	na	111.2 ^a^	109.6 ^a^	105.8 ^a^	111.1 ^a^
pH	na	na	na	3.8 ^a^	3.8 ^a^	3.8 ^a^	3.8 ^a^
Reducing sugars (g L^−1^)	na	na	na	3.3 ^a^	3.2 ^a^	3.3 ^a^	3.2 ^a^
Reduced dry extract (g L^−1^)	na	na	na	27.1 ^a^	27.1 ^a^	27.3 ^a^	27.4 ^a^
Total phenolic and flavonoid content and antioxidant capacity							
TPC (mg L^−1^ GAE)	1965.0 ^b^	1193.3 ^c^	2843.3 ^a^	459.4 ^c^	481.9 ^b^	474.3 ^b^	495.2 ^a^
TFC (mg L^−1^ CAE)	347.2 ^b^	216.2 ^c^	1247.0 ^a^	32.1 ^d^	41.4 ^b^	35.9 ^c^	64.4 ^a^
DPPH (IC_50_)	119.7 ^b^	223.6 ^a^	44.3 ^c^	26.9 ^b^	28.0 ^a^	25.2 ^c^	22.4 ^d^
ORAC (µmol L^−1^ TE)	2.5 ^a^	2.6 ^a^	2.6 ^a^	1.6 ^b^	1.8 ^b^	1.9 ^b^	2.2 ^a^

MR = *Matricaria recutita* L. (chamomile); CC = *Cymbopogon citratus* (lemongrass); MP = *Mentha piperita* L. (peppermint); na = not analyzed; mEq = milliequivalent; TPC = total phenolic content; GAE = gallic acid equivalent; TFC = total flavonoid content; CAE = catechin equivalent; DPPH = 2,2-diphenyl-1-picril-hydrazine; IC_50_ = sample volume (μL) to remove 50% of DPPH; ORAC = oxygen radical absorbance capacity; TE = Trolox equivalent. Different lower-case letters, in extract or in fermented beverage, indicate means with significant differences (*p* < 0.05) by the Tukey test.

**Table 3:** Phenolic composition of the functional fermented orange beverage containing aromatic herbal extracts.

Parameters	RT (min)	Crude Extract			Fermented Orange Beverage			
		MR	CC	MP	Control	Fermented + MR	Fermented + CC	Fermented + MP
Phenolic acids								
Chlorogenic acid (mg L^−1^)	15.1	49.1 ^a^	8.4 ^c^	47.4 ^b^	10.9 ^c^	16.0 ^a^	13.8 ^b^	10.8 ^c^
Caffeic acid (mg L^−1^)	16.5	nd	3.5	nd	5.3 ^a^	5.5 ^a^	5.3 ^a^	5.3 ^a^
Ferulic acid (mg L^−1^)	23.2	95.2 ^b^	nd	98.7 ^a^	1.6 ^ab^	1.7 ^a^	1.0 ^c^	1.1 ^bc^
Flavonoids								
Narirutin (mg L^−1^)	26.1	nd	nd	nd	69.7 ^a^	70.6 ^a^	66.5 ^b^	69.6 ^a^
Rutin (mg L^−1^)	27.1	nd	nd	nd	6.1	nd	nd	nd
Hesperidin (mg L^−1^)	28.1	nd	nd	nd	130.3 ^a^	129.9 ^a^	124.3 ^b^	125.1 ^b^
Luteolin (mg L^−1^)	37.3	20.1 ^a^	3.1 ^c^	17.4 ^b^	nd	nd	nd	nd
Apigenin (mg L^−1^)	39.2	138.4 ^a^	nd	132.4 ^a^	nd	nd	nd	nd

MR = *Matricaria recutita* L. (chamomile); CC = *Cymbopogon citratus* (lemongrass); MP = *Mentha piperita* L. (peppermint); nd = not detected. Different letters, in extract or in fermented beverage, indicate means with significant differences (*p* < 0.05) by the Tukey test.

**Table 4:** In silico prediction of toxicity and biological activity based on the major phenolic compounds present in fermented orange beverage.

	In Silico	Chlorogenic Acid	Caffeic Acid	Ferulic Acid	Narirutin	Rutin	Hesperidin
Prediction of toxicity	Mutagenic (AMES toxicity)	(-)^pkCSM^ (-)^admetSAR^ (-)^Protox^ (-)^Lazar^	(-)^pkCSM^ (-)^admetSAR^ (-)^Protox ^ (-)^Lazar^	(-)^pkCSM^ (-)^admetSAR^ (-)^Protox^ (-)^Lazar^	(-)^pkCSM^ (-)^admetSAR^ (-)^Protox^ (-)^Lazar^	(-)^pkCSM^ (-)^admetSAR^ (-)^Protox^	(-)^pkCSM^ (-)^admetSAR^ (-)^Protox^
	Carcinogenic	(-)^admetSAR^ (-)^Protox^ (-)^Lazar^	(-)^admetSAR^ (-)^Lazar^	(-)^admetSAR^ (-)^Protox^ (-)^Lazar^	(-)^pkCSM^ (-)^admetSAR^ (-)^Protox^	(-)^pkCSM^ (-)^admetSAR^ (-)^Protox^	(-) ^pkCSM^ (-)^admetSAR^ (-)^Protox^
	Hepatotoxicity	(-)^pkCSM^	(-)^pkCSM^ (-)^Protox^	(-)^pkCSM^ (-) ^Protox^	(-)^pkCSM^ (-) ^Protox^	(-)^pkCSM^ (-) ^Protox^	(-)^pkCSM^ (-) ^Protox^
	Blood–brain barrier penetration	(-)^pkCSM^ (-)^Lazar^	(-)^pkCSM^ (-)^admetSAR^ (-)^Lazar^	(-)^pkCSM^ (-)^admetSAR^ (-)^Lazar^	(-)^pkCSM^ (-)^admetSAR^ (-)^Lazar^	(-)^pkCSM^ (-)^admetSAR^ (-)^Lazar^	(-)^pkCSM^ (-)^admetSAR^ (-)^Lazar^
	Acute oral toxicity	* III ^admetSAR^	* IV ^admetSAR^	* IV ^admetSAR^	* III ^admetSAR^	* III ^admetSAR^	* III ^admetSAR^
Prediction of biological activity	Antihemorrhagic	Pa ^PASS (16.4%)^	n.r.	n.r.	Pa ^PASS (81.5%)^	Pa ^PASS (90.4%)^	Pa ^PASS (72.7%)^
	Antioxidant	Pa ^PASS (80.9%)^	Pa ^PASS (61.1%)^	Pa ^PASS (54.7%)^	Pa ^PASS (88.0%)^	Pa ^PASS (92.7%)^	Pa ^PASS (85.3%)^
	Free radical scavenging	Pa ^PASS (85.6%)^	Pa ^PASS (67.0%)^	Pa ^PASS (74.1%)^	Pa ^PASS (98.2%)^	Pa ^PASS (99.0%)^	Pa ^PASS (99.1%)^
	Anti-inflammatory	Pa ^PASS (65.7%)^	Pa ^PASS (64.8%)^	Pa ^PASS (66.1%)^	Pa ^PASS (71.6%)^	Pa ^PASS (74.6%)^	Pa ^PASS (70.4%)^
	Antiulcerative	Pa ^PASS (54.2%)^	Pa ^PASS (61.0%)^	Pa ^PASS (60.4%)^	Pa ^PASS (71.6%)^	Pa ^PASS (58.5%)^	Pa ^PASS (70,9%)^
	Gastritis treatment	Pa ^PASS (27.1%)^	Pa ^PASS (35.5%)^	Pa ^PASS (38.4%)^	Pa ^PASS (35.5%)^	Pa ^PASS (49.6%)^	Pa ^PASS (39,9%)^
	Mucomembranous protection	Pa ^PASS (75.2%)^	Pa ^PASS (94.5%)^	Pa ^PASS (90.6%)^	n.r.	n.r.	n.r.
	Vasoprotection	Pa ^PASS (44.2%)^	Pa ^PASS (78.2%)^	Pa ^PASS (75.3%)^	Pa^PASS (97.0%)^	Pa ^PASS (98.0%)^	Pa ^PASS (97.4%)^

* LD_50_ compounds are classified into four categories based on US EPA. (Category I: compounds with LD_50_ values ≤ 50 mg kg^−1^. Category II: compounds with LD_50_ values > 50mg kg^−1^ and < 500mg kg^−1^. Category III: compounds with LD_50_ values ≥ 500 mg kg^−1^ and < 5000 mg kg^−1^. Category IV: compounds with LD_50_ values ≥ 5000 mg kg^−1^ and < 5000 mg kg^−1^) (+) = at risk; (-) = not at risk; (n.r.) = not reported; Pa = probably active; Pi = probably inactive.

## Data Availability

No new data were created or analyzed in this study. Data sharing is not applicable to this article.

## Supplementary Materials

The following supporting information can be downloaded at: https://www.mdpi.com/article/10.3390/foods12020243/s1, Figure S1: Representative chromatogram of fermented orange beverage acquired at 280 nm; Figure S2: Representative chromatogram of *Matricaria recutita* L extract acquired at 280 nm; Figure S3: Representative chromatogram of *Cymbopogon citratus* extract acquired at 280 nm; Figure S4: Representative chromatogram of *Mentha piperita* extract acquired at 280 nm.
