# Supplementary material for: Effect of Adding Matricaria recutita L., Cymbopogon citratus, or Mentha piperita L. Extracts to Fermented Orange Beverage: Sensory Evaluation, Physicochemical Characterization, and Prediction of Toxic Risks and Biological Activity In Silico
Source: Foods. 2023 Jan 5;12(2):243. doi: 10.3390/foods12020243 (PMC9858046; doi:10.3390/foods12020243)
Supplement: Supplementary file 1 [file foods-12-00243-s001.zip › Supplementary material.pdf]

foods

Suplementary Material

Effect of Adding *Matricaria recutita* L., *Cymbopogon citratus*, or *Mentha piperita* L. Extracts on Fermented Orange Beverage: Sensory Evaluation, Physicochemical Characterization, and Prediction of Toxic Risks and Biological Activity *In Silico*

Laura Gizele Mascarin [1], Fernanda Wouters Franco [1], Rafaela Castro Dornelles [2], Kássia Caroline Figueredo [2], Roberta Oliveira Santos [3], Liliane de Freitas Bauermann [2], Tatiana Emanuelli [1], Sabrina Somacal [1,*], Cláudia Kaehler Sautter [1]

Abstract: Fermentation is an important tool in producing functional beverages through agro-industrial wastes and medicinal and aromatic plants due to the specific content of bioactive molecules. Therefore, this study evaluated the contribution of *Matricaria recutita* (chamomile), *Cymbopogon citratus* (lemongrass), or *Mentha piperita* (peppermint) extracts to phytochemical profile and potential biological effects of a functional orange fermented beverage *in vitro* and *in silico*. The concentrations of aromatic herbal extracts that yielded the best sensory performance for fermented beverages were selected for analyses that involved characterizing the fermented beverages. The beverages that received the extracts (2%) had the highest phenolic and flavonoid content and antioxidant potential compared to the control. Hesperidin (124–130 mg L^{-1}), narirutin (66–70 mg L^{-1}), chlorogenic (11–16 mg L^{-1}), caffeic (5.3–5.5 mg L^{-1}), and ferulic (1–1.7 mg L^{-1}) acids were found in the different formulation. The *in silico* analysis suggested that the evaluated compounds do not present toxicity risk (mutagenicity, carcinogenicity, hepatotoxicity, and ability to penetrate the blood-brain barrier). Additionally, it can contribute to the biological effects of therapeutic importance such as antioxidants, gastroprotective, and anti-ulcerative properties, and the *Mentha piperita* L. extract presented the greatest potential among the evaluated herbs for use in functional fermented beverages.

Keywords: antioxidants; *Cymbopogon citratus*; fermented beverage; functional beverage; gastroprotective; *Matricaria recutita* L.; *Mentha piperita* L.; phenolic compounds

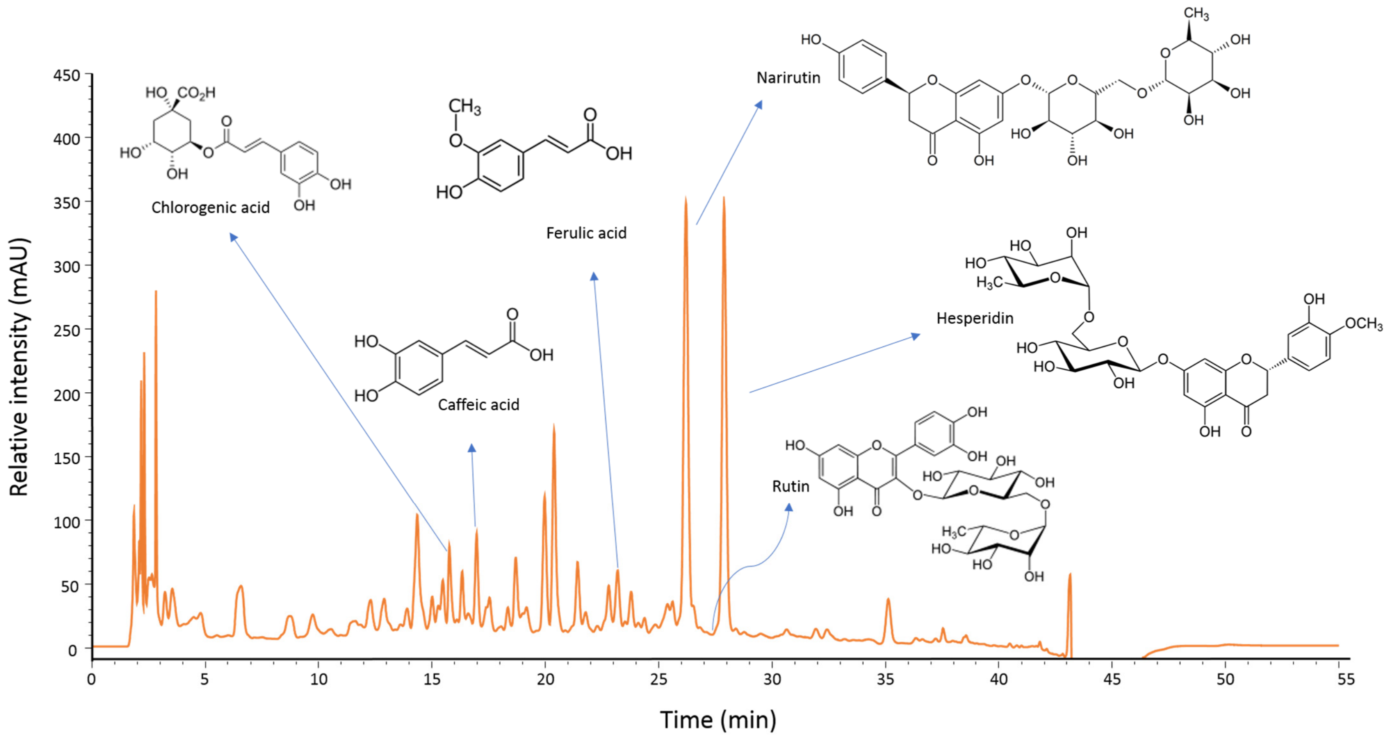

Figure S1. Representative chromatogram of fermented orange beverage acquired at 280 nm

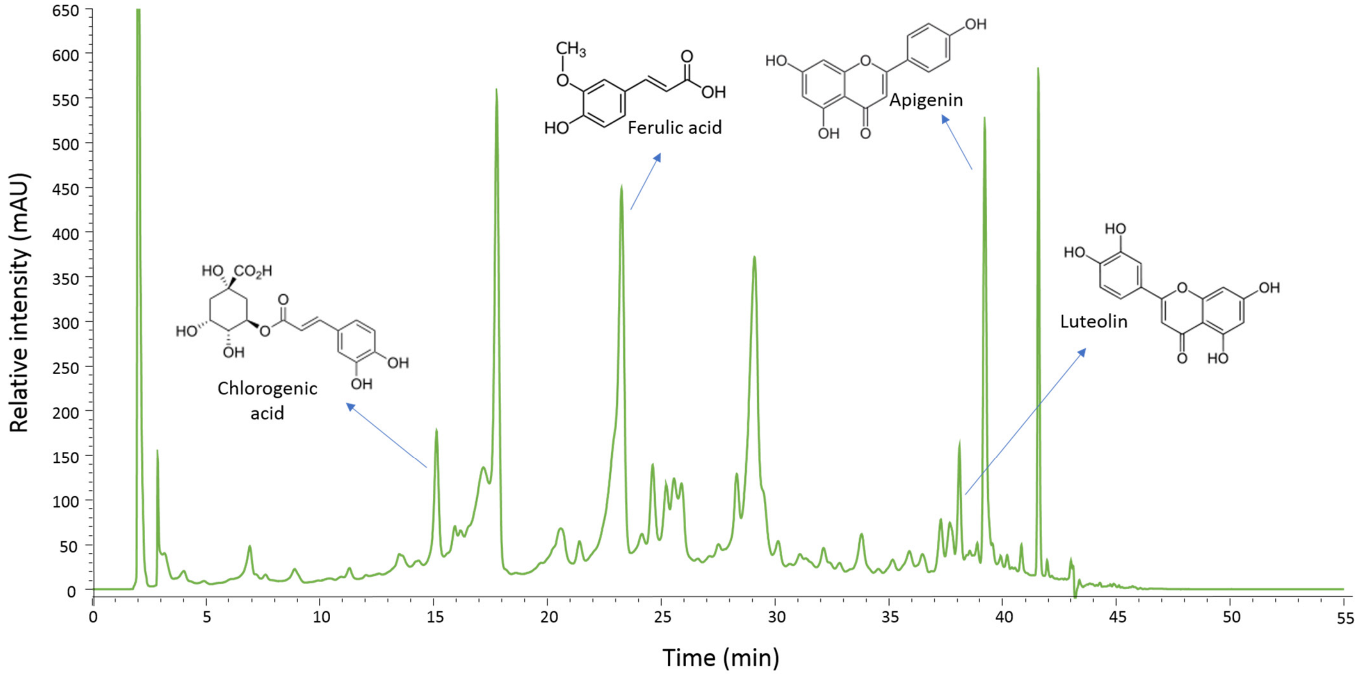

Figure S2. Representative chromatogram of *Matricaria recutita* L extract acquired at 280 nm

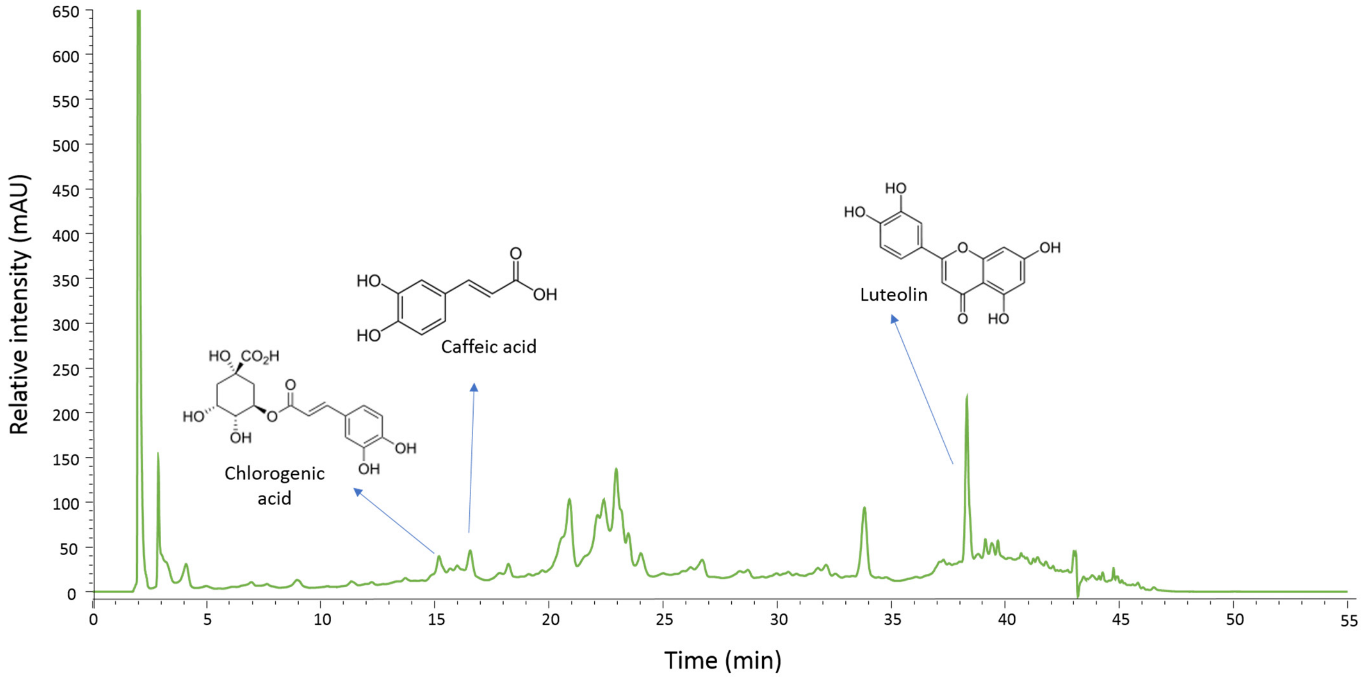

Figure S3. Representative chromatogram of *Cymbopogon citratus* extract acquired at 280 nm

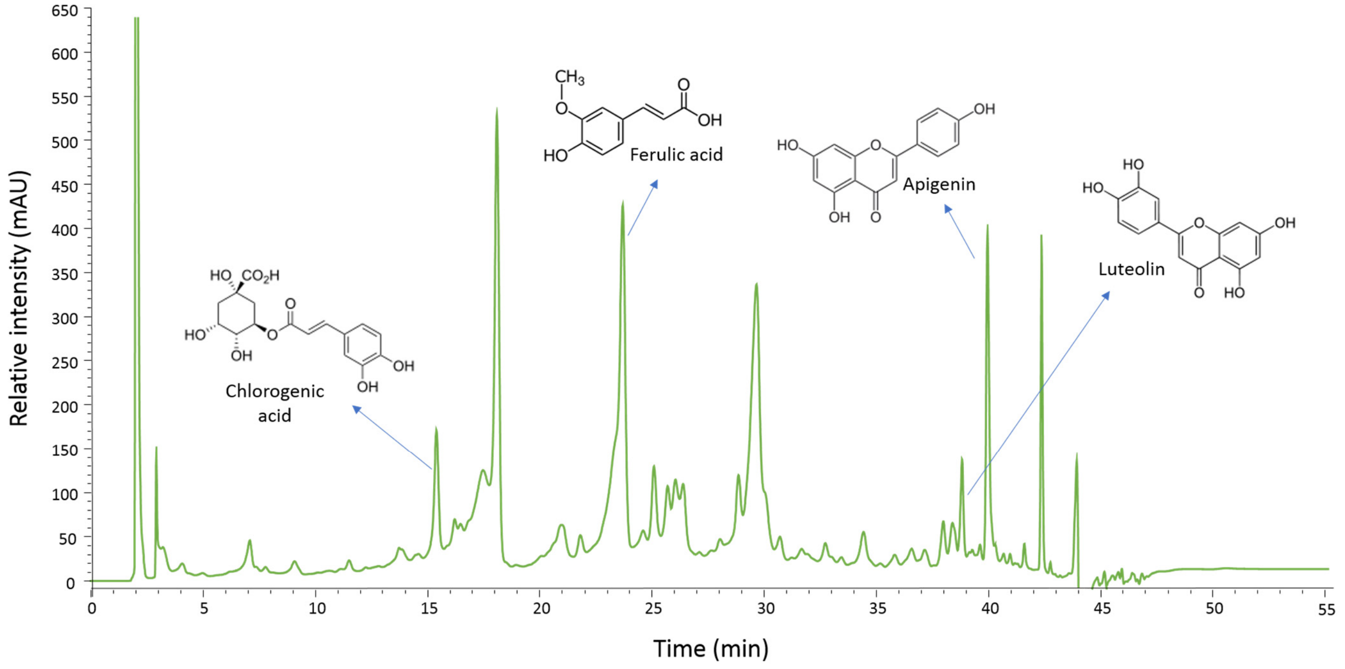

Figure S4. Representative chromatogram of *Mentha piperita* extract acquired at 280 nm

PARECER CONSUBSTANCIADO DO CEP

DADOS DO PROJETO DE PESQUISA

Título da Pesquisa: Fermentado de laranja composto de extrato de ervas aromáticas: desenvolvimento, caracterização e avaliação do potencial antioxidante e gastroprotetor.

Pesquisador: CLÁUDIA KAEHLER SAUTTER

Área Temática:

Versão: 1

CAAE: 10027519.3.0000.5346

Instituição Proponente: Universidade Federal de Santa Maria

Patrocinador Principal: Financiamento Próprio

DADOS DO PARECER

Número do Parecer: 3.257.616

Apresentação do Projeto:

O projeto busca o desenvolvimento e a caracterização de uma bebida fermentada a partir de laranja acrescida de extratos de ervas aromáticas (capim limão, camomila e hortelã).

É um projeto de doutorado apresentado para o curso de Ciência dos Alimentos Tem uma introdução que descreve sobre o consumo de bebida alcóolica, fermentados e vinhos, seus benefícios bem como sua relação com a formação de úlceras gástricas. Em seguida o autor aborda o uso de extratos de ervas aromáticas em bebidas alcoólicas e seus benefícios para a saúde.

Será feito um fermentado de laranja (coletadas na cidade de Mata) e a este será acrescido extratos das ervas, mantidos em ambiente 5ºC/48 horas para decantação das leveduras e outros sólidos e incorporação/extração de compostos. Após, será realizada a trasfega, filtração e clarificação e acondicionamento. Esses produtos serão submetidos a avaliação sensorial, através do teste de aceitabilidade de Dutcoski. Os sujeitos a avaliar a bebida serão estudantes e funcionários da UFSM (100 julgadores). Os dados de fatores de inclusão e exclusão são descritos também.

No projeto possui um item para descrever os aspectos éticos, contendo a descrição dos riscos e benefícios.

Serão feitas análises físico-quimica, determinação da capacidade antioxidante, estudos com animais serão realizados após aprovação do projeto no CEUA.

No projeto consta um orçamento e cronograma em acordo com as exigências do CEP.

Objetivo da Pesquisa:

OBJETIVO GERAL: caracterizar um fermentado de laranja (Citrus sp.) composto de extrato de ervas aromáticas com potencial antioxidante e gastroprotetor.

OBJETIVOS ESPECÍFICOS

• Desenvolver e caracterizar um fermentado de laranja composto;

• Desenvolver e caracterizar extratos hidro alcoólicos macerados a partir de flores de Camomila (Chamomila recutita L.), folhas de Capim-limão (Cymbopogon citratus) e folhas de Hortelã (Mentha piperita L.);

• Eleger as concentrações de extratos a serem utilizadas no fermentado final, através da análise sensorial: teste de aceitabilidade, teste de ordenação-preferência e teste de intenção de compra.

• Aplicar os extratos hidro alcoólicos macerados de camomila, capim-limão e hortelã no fermentado de laranja (tratamentos: T1 = laranja, T2 = laranja + camomila, T3 = laranja + capim-limão, T4 = laranja + hortelã) e avaliar a capacidade antioxidante dos tratamentos do fermentado de laranja composto;

• Testar in vivo a possível capacidade gastroprotetora (proteção ou reparação de dano) do fermentado alcoólico de laranja e seus tratamentos (T1, T2, T3 e T4), frente à úlcera gástrica induzida por etanol.

Avaliação dos Riscos e Benefícios:

Os provadores serão informados que a pesquisa trará maior conhecimento sobre o tema abordado, sem benefício direto.

Os riscos para a realização desta degustação serão considerados mínimos, pois a bebida desenvolvida utiliza em sua formulação ingredientes destinados para fins alimentícios e o volume de amostra a ser provado é pequeno. Porém, há que se considerar os efeitos do consumo de bebida alcoólica, como tontura, dor de cabeça, mal-estar geral, diminuição dos reflexos e coordenação motora, além de quadros alérgicos que possam se desenvolver em indivíduos com

hipersensibilidade a algum componente da bebida ou dos extratos das plantas. Caso algo ocorra, o participante deve entrar em contato com os responsáveis desse projeto a fim de ser encaminhado para a assistência médica.

Comentários e Considerações sobre a Pesquisa:

.

Considerações sobre os Termos de apresentação obrigatória:

Os termos são suficientes e atendem a legislação.

Recomendações:

.

Conclusões ou Pendências e Lista de Inadequações:

.

Considerações Finais a critério do CEP:

Este parecer foi elaborado baseado nos documentos abaixo relacionados:

Tipo Documento	Arquivo	Postagem	Autor	Situação
Informações Básicas do Projeto	PB_INFORMAÇÕES_BÁSICAS_DO_PROJETO_1305464.pdf	19/03/2019 15:41:35		Aceito
Outros	TCLE_Corrigido.pdf	19/03/2019 15:41:03	CLÁUDIA KAEHLER SAUTTER	Aceito
Outros	Projeto_GAP_045196_Fermentado_de_laranja.pdf	19/03/2019 15:27:21	CLÁUDIA KAEHLER SAUTTER	Aceito
Outros	Autorizacao_institucional.pdf	28/02/2019 11:34:58	CLÁUDIA KAEHLER SAUTTER	Aceito
Outros	Termo_de_Confidencialidade.pdf	28/02/2019 11:34:11	CLÁUDIA KAEHLER SAUTTER	Aceito
TCLE / Termos de Assentimento / Justificativa de Ausência	Termo_Livre_Esclarecido.pdf	28/02/2019 11:33:36	CLÁUDIA KAEHLER SAUTTER	Aceito
Projeto Detalhado / Brochura	Projeto_Sensorial.pdf	28/02/2019 11:33:09	CLÁUDIA KAEHLER SAUTTER	Aceito

Investigador	Projeto_Sensorial.pdf	28/02/2019 11:33:09	CLÁUDIA KAEHLER SAUTTER	Aceito
Folha de Rosto	Folha_de_Rosto.pdf	28/02/2019 11:25:34	CLÁUDIA KAEHLER SAUTTER	Aceito

Situação do Parecer:
Aprovado

Necessita Apreciação da CONEP:
Não

SANTA MARIA, 10 de Abril de 2019

Assinado por:
CLAUDEMIR DE QUADROS
(Coordenador(a))
